# Autochthonous Rat Lungworm *Angiostrongylus cantonensis* Infections in Accidental and Definitive Hosts, San Diego, California, USA

**DOI:** 10.3201/eid3202.251081

**Published:** 2026-02

**Authors:** Shotaro Nakagun, Carlo G. Gonzalez Vera, Alexis Wohl, Deana L. Clifford, Garrett A. Fraess, Jordyn R. Nylander, Estefanía Montero, Javier Asin, Steven V. Kubiski, Rachel E. Burns

**Affiliations:** Author affiliations: San Diego Zoo Wildlife Alliance, San Diego, California, USA (S. Nakagun, C.G. Gonzalez Vera, G.A. Fraess, J.R. Nylander, S.V. Kubiski, R.E. Burns); San Diego Humane Society’s Project Wildlife, San Diego (A. Wohl); California Department of Fish and Wildlife, Rancho Cordova, California, USA (D.L. Clifford); Universidad Cardenal Herrera-CEU, CEU Universities, Valencia, Spain (E. Montero); California Animal Health and Food Safety Laboratory, University of California, Davis, San Bernardino, California, USA (J. Asin)

**Keywords:** angiostrongyliasis, *Angiostrongylus cantonensis*, rat lungworm, parasites, meningitis/encephalitis, zoonoses, accidental host, autochthonous infection, nematode, rat, United States

## Abstract

The rat lungworm, *Angiostrongylus cantonensis*, is an emerging veterinary and public health concern. We describe *A. cantonensis* infections in a zoo-housed parma wallaby and free-ranging Virginia opossums and roof rats in San Diego, California, USA. Angiostrongyliasis should be considered in central nervous system disease in humans and animals in this region.

*Angiostrongylus cantonensis*, the rat lungworm, is an invasive, zoonotic metastrongyle nematode that causes neurologic disease in humans and other vertebrate hosts (*1*). The usual life cycle of this parasite involves infection of the rodent definitive host by ingesting third-stage larvae (L3) found within gastropod intermediate hosts. Less commonly, paratenic hosts such as frogs, lizards, and crustaceans harbor the infective L3 and transfer *A. cantonensis* lungworm to rodents and accidental or aberrant hosts (*1*).

Since the original discovery of *A. cantonensis* lungworm in southern China in 1935 (*2*), the nematode has spread globally, including to Hawaii and more recently, the southeastern United States. In the continental United States, its range has gradually spread; infections in free-ranging rodents, gastropods, or both have been confirmed in Louisiana (*3*), Florida (*4*), Oklahoma (*5*), Georgia (*6**–**7*), and Texas (*8*). Hence, *A. cantonensis* lungworm is now considered endemic in those states. Cases in human and nonhuman accidental hosts have been reported in many more continental US states, with autochthonous infections suspected in Alabama (*9*), Mississippi (*10*), and Tennessee (*11*). In California, human cases have been sporadically reported in those who had traveled to endemic areas outside the continental United States and in those with unknown travel history (*12*). We describe *A. cantonensis* infections in a zoo-housed parma wallaby (*Notamacropus parma*) and free-ranging Virginia opossums (*Didelphis virginiana*) and roof rats (*Rattus rattus*) in San Diego, California, USA.

## The Study

**Figure 1:**
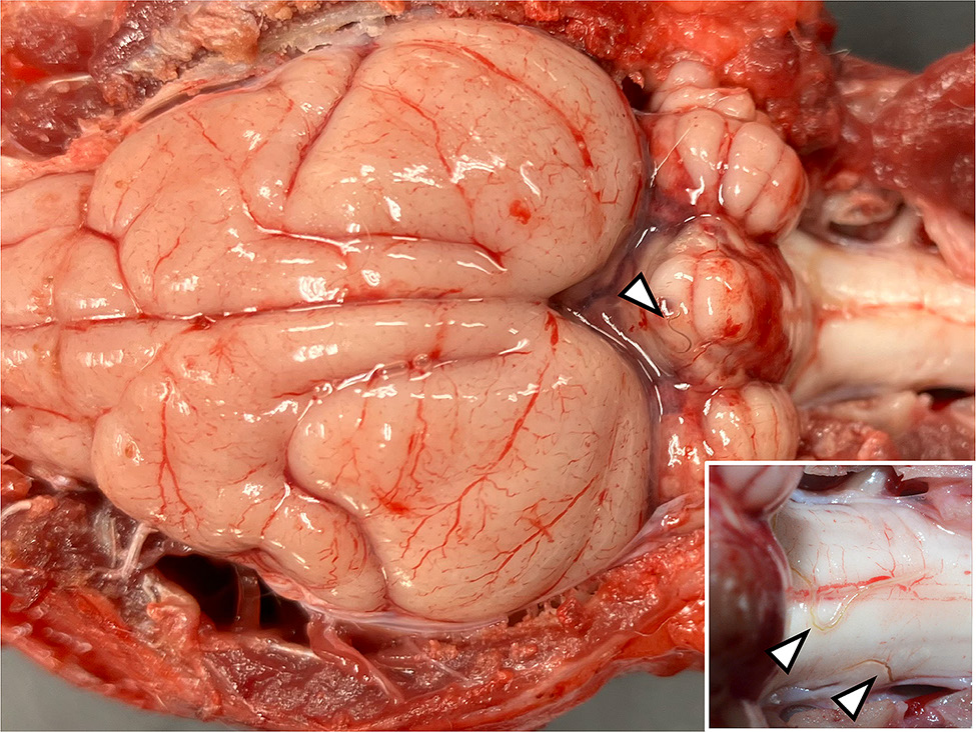
Brain and cervical spinal cord tissue from a parma wallaby (*Notamacropus parma*) with autochthonous rat lungworm *Angiostrongylus cantonensis* infection, San Diego, California, USA, 2024. The dorsal and caudal aspects of the cerebellum have coalescing foci of hemorrhage with nematodes (arrows) on the leptomeningeal surface. The inset depicts an enlarged view of the dorsal aspect of brainstem and C1 spinal cord with visible nematodes (arrows).

In mid-December 2024, a 7-year-old male parma wallaby that was born and raised at the San Diego Zoo (San Diego, CA, USA) showed progressive neurologic signs, including head shaking, nystagmus, central blindness, ataxia, hindlimb paresis, and extensor rigidity in all limbs. The wallaby was euthanized after 11 days of hospitalization. Necropsy revealed multifocal hemorrhage in the cerebellum and half a dozen nematodes on the leptomeningeal surfaces of the cerebellum, brainstem, and cervical spinal cord (Figure 1). Histopathology revealed necrotizing and lymphoplasmacytic meningoencephalomyelitis with fibrinoid vascular necrosis, infarction, and hemorrhage, most severely affecting the cerebellum and right cerebrum. The lesions were occasionally associated with intralesional and rarely intraparenchymal metastrongyle nematodes. A nematode collected in 70% ethanol was identified as *A. cantonensis* lungworm through PCR and sequencing (Appendix).

**Figure 2:**
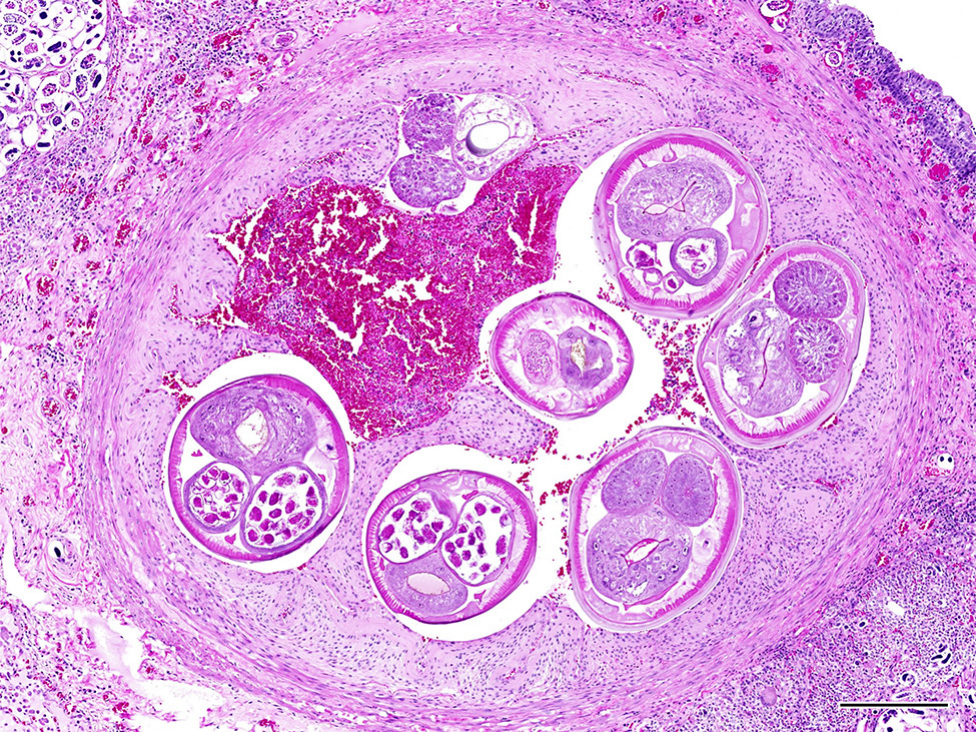
Lung tissue from a roof rat (*Rattus rattus*) with autochthonous rat lungworm *Angiostrongylus cantonensis* infection, San Diego, California, USA, 2025. Hematoxylin and eosin stain of pulmonary arteries with intravascular adult metastrongyle nematodes have severe endothelial proliferation. The surrounding lung parenchyma is replaced by granulomatous inflammation centered on numerous larvated and morulated eggs. Scale bar indicates 250 µm.

Because the diagnosis was unusual in this geographic area (*3*–*11*), we performed necropsies on free-ranging roof rats that were either euthanized as part of regular pest control or found dead on the San Diego Zoo grounds during January 14–February 14, 2025. We grossly examined 64 rats comprising both sexes and all life stages and evaluated lung and feces samples histologically and by the Baermann technique for all rats, regardless of gross findings. Two (3.1%) adult rats had lungworms and associated pneumonia. Histologically, the main findings were pulmonary arterial endothelial proliferation with thrombosis and adult metastrongyle nematodes (Figure 2) and granulomatous and fibrinosuppurative to fibrosing pneumonia with myriad intralesional adult and larval metastrongyle nematodes and eggs. Fecal examination of the 2 affected rats revealed numerous live, ≈300-μm–long larvae with coiled posterior ends. Molecular analyses of ethanol-fixed adult nematodes within the pulmonary artery and fresh feces revealed genetic sequences of *A. cantonensis* lungworm.

**Figure 3:**
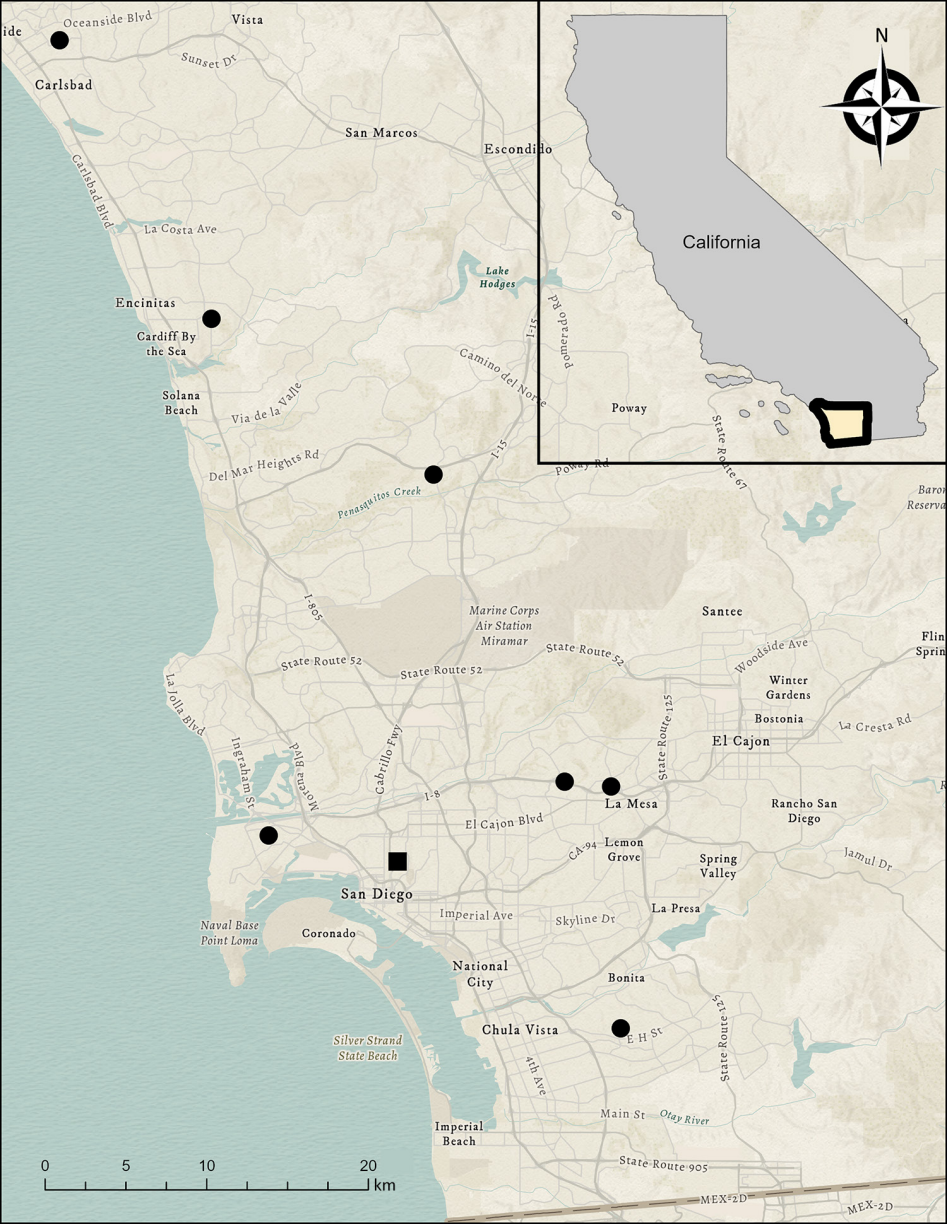
Distribution of Virginia opossums (*Didelphis virginiana*) identified with autochthonous rat lungworm *Angiostrongylus cantonensis* infections, San Diego, California, USA, 2023–2025. Black dots indicate opossum cases, which ranged over ≈68 km from north to south. The location of the San Diego Zoo, where cases of angiostrongyliasis were identified in a wallaby and rats, is included for reference (black square). Inset shows location of study area in California. Sources: Airbus Defence and Space, CGIAR, Danish Geodata Agency, Esri, Food and Agriculture Organization of the United Nations, Federal Emergency Management Agency, Garmin, General Services Admission, Geoland, Intermap, National Aeronautics and Space Administration, National Center for Ecological Analysis and Synthesis, National Geospatial-Intelligence Agency, National Land Service, National Mapping Agency, National Oceanic and Atmospheric Administration, ©OpenStreetMap contributors, Ordnance Survey, Rijkswaterstaat, Robinson Projection, TomTom, US Geological Society, Vantor, and the Geographic Information Systems user community.

Concurrently, a local wildlife rehabilitation program had received multiple free-ranging juvenile Virginia opossums from throughout San Diego County (Figure 3) that exhibited >1 clinical signs, including dull mentation, circling, head tilt, ataxia, and respiratory distress. Most of the opossums were received during June–September in 2023–2025 and were euthanized after 0–3 days because of lack of clinical improvement. Ten opossums were submitted for postmortem examination. A consistent finding in 7 opossums was eosinophilic meningitis with intralesional metastrongyle nematodes (Appendix Figure), whereas 1 animal had eosinophilic meningitis without nematodes. The identity of the nematodes was confirmed as *A. cantonensis* lungworm in 6 of 7 animals through molecular analyses on formalin-fixed paraffin-embedded brain tissue (Appendix). We deposited generated nucleotide sequences from this study into GenBank (Table). 

**Table:** Clinical and molecular information for animals with autochthonous rat lungworm *Angiostrongylus cantonensis* infections, San Diego, California, USA, 2023–2025*

Case ID	Species	Sex	Age class	Death date	GenBank accession no.		
					COI	28S rRNA	18S rRNA
1	Parma wallaby	M	Adult	2024 Dec 17	PX623034	PX623182	PV933159
2	Roof rat	F	Adult	2025 Feb 3	PX623032	PX623180	PV933160
3	Roof rat	M	Adult	2025 Feb 5	PX623033	PX623181	PV933161
4	Virginia opossum	M	Juvenile	2023 Aug 27	PX623045	PX623179	NT
5	Virginia opossum	M	Juvenile	2025 Jun 27	PX623044	PX623178	NT
6	Virginia opossum	F	Juvenile	2025 Aug 15	NA†	PX663655	NT
7	Virginia opossum	F	Juvenile	2025 Sep 6	NA†	NA†	NT
8	Virginia opossum	M	Juvenile	2025 Sep 7	Partial‡	PX623200	NT
9	Virginia opossum	M	Juvenile	2025 Sep 7	Partial‡	PX663656	NT
10	Virginia opossum	M	Juvenile	2025 Sep 12	NA†	PX623201	NT

*ID, identification; COI, cytochrome C oxidase subunit I gene; NA, no amplification; NT, not tested. †Lack of specific amplification after multiple PCR and sequencing attempts was attributed to the sample type (formalin-fixed paraffin embedded tissue). ‡Obtained partial sequences were too short to be deposited in GenBank.

In addition, in an effort to investigate *A. cantonensis* lungworm in intermediate hosts, we opportunistically collected free-ranging slugs from the wallaby enclosure and adjacent areas at the San Diego Zoo (Appendix). One slug had nematode larvae encysted in various tissues, but we were unable to confirm the species identity.

## Conclusions

We documented *A*. *cantonensis* infections in a zoo-housed parma wallaby (accidental host), free-ranging Virginia opossums (accidental host), and free-ranging roof rats (definitive host) in San Diego, California. Most cases were identified in 2025, but infection occurred as early as August 2023 in 1 opossum. Our findings indicate autochthonous infections, which pose a substantial risk to humans and accidental vertebrate hosts. Whereas autochthonous infections had not previously been documented in the United States west of Texas, identifying angiostrongyliasis cases in wildlife in San Diego County provides support that *A*. *cantonensis* lungworm could now be considered endemic in this portion of southern California, with the potential to spread to other parts of the western continental United States.

Identifying the source of introduction of *A. cantonensis* lungworm into San Diego was beyond the scope of this study. In Louisiana, where the nematode was first identified in the continental United States in 1987, introduction was suspected to have been from infected rats on ships that docked in New Orleans (*3*). A similar situation is possible in this instance, given that San Diego is a major port city. However, the volume of traffic between regions on both national and international scales has dramatically expanded since the 1980s; thus, introduction of invasive species to new areas is a constant threat (*13*). A likely additional factor is the distributional spread of invasive gastropods (*4**,**8*) (Appendix).

Infections in the zoo-housed wallaby and free-ranging opossums were likely the result of ingestion of an infected intermediate host. The wallaby was fed a commercially manufactured extruded diet with a mixture of grass hay and occasional browse material and fresh produce safe for human consumption; ingestion of an intermediate host was considered accidental environmental exposure. In contrast, opossums are naturally more vulnerable to infection, given that gastropods constitute a prominent part of their native diet, especially during the summer months (*14*). All the opossums identified with angiostrongyliasis in our study were juveniles found in the summer, suggesting the juvenile age group and summer season are risk factors for infection in this species.

*A. cantonensis* infection has previous been identified at the San Diego Zoo. In 2011, an African pygmy falcon (*Polihierax semitorquatus*) hatched and raised at the San Diego Zoo was diagnosed with meningoencephalitis caused by *A. cantonensis* infection (*15*). At that time, 20 free-ranging rats on the zoo grounds were opportunistically screened for nematode larvae in feces, but infection was not detected. With no detection in definitive hosts, the source of infection in that case and in 2 subsequent African pygmy falcons in 2014 and 2018 (San Diego Zoo, unpub. data) was suspected to be live feeder lizards (paratenic hosts) imported from Southeast Asia, where the parasite is endemic. However, given the prevalence (albeit low at 3.1%) of infected rats in our study, the parasite possibly has been present but undetected in San Diego County for some time.

In conclusion, we documented autochthonous *A. cantonensis* infections in southern California, highlighting a notable expansion of the range of this parasite in North America. Further studies are needed to analyze the effect of this geographic expansion and associated risks in California. Nevertheless, angiostrongyliasis should be part of the differential diagnosis for central nervous system disease in humans and animals in the wider southern United States. 

Appendix Additional information for autochthonous rat lungworm *Angiostrongylus cantonensis* infections in accidental and definitive hosts, San Diego, California, USA.
