# Supplementary material for: Autochthonous Rat Lungworm Angiostrongylus cantonensis Infections in Accidental and Definitive Hosts, San Diego, California, USA
Source: Emerg Infect Dis. 2026 Feb;32(2):265–9. doi: 10.3201/eid3202.251081 (PMC12928223; doi:10.3201/eid3202.251081)
Supplement: Appendix — Additional information for autochthonous rat lungworm Angiostrongylus cantonensis infections in accidental and definitive hosts, San Diego, California, USA. [file 25-1081-Techapp-s1.pdf]

Article DOI: https://doi.org/10.3201/eid3202.251081

EID cannot ensure accessibility for supplementary materials supplied by authors. Readers who have difficulty accessing supplementary content should contact the authors for assistance.

Autochthonous Rat Lungworm *Angiostrongylus cantonensis* Infections in Accidental and Definitive Hosts, San Diego, California, USA

Appendix

Molecular Testing Methods

Genomic nematode DNA was extracted from ethanol-fixed specimens or formalin-fixed paraffin-embedded tissue using DNeasy Blood & Tissue Kit (QIAGEN) and QIAamp DNA FFPE Tissue Kit (QIAGEN), respectively, following the manufacturer's recommendations. The tissues were pretreated with mechanical bead beating using the TissueLyser III (QIAGEN) with 1.0 mm silicon carbide beads for 1 minute. For feces, genomic DNA was extracted using DNeasy PowerSoil Pro Kit (QIAGEN) following the manufacturer's recommendations.

PCR amplification of the cytochrome c oxidase subunit I (COI) gene was carried out following a protocol described previously (*1*,*2*). Amplification of the 18S ribosomal RNA (rRNA) gene was similarly performed following protocols described previously (*3*), only on DNA extracted from nematodes fixed in ethanol and feces. Amplification of the 28S rRNA gene was completed using the following primers: LSU370F (5′-ACGAGATTCCCACTGTCCCTAACTAC-3′) and LSU765R (5′-GCTTTTGCCCTTTTGCTCTACGA-3′) with PCR conditions consisting of an initial denaturation at 95°C for 5 min, followed by 40 cycles of denaturation at 95°C for 50 sec, annealing at 55°C for 1 min, and extension at 72°C for 30 sec, and a final extension at 72°C for 5 min. All PCR reactions used 25 µL PCR mixtures containing 0.4 µM of each primer and 1X MyTaq Red Mix (Bioline). Amplification was performed using a T100 thermal cycler (BioRad). PCR products were detected on 1.5% gels stained with ethidium bromide. Products of expected

size (COI, 450 bp (*1*) or 605 bp (*2*); 18S rRNA, 1.1 kbp; 28S rRNA, 393 bp) were cut out of the gels and purified using Ultrafree-DA Centrifugal Filters (Millipore) followed by sanger sequencing. Consensus sequences were generated from both strands to be aligned and compared with homologous sequences of *Angiostrongylus cantonensis* available in GenBank. Nucleotide sequences generated in this study were deposited in GenBank under accession numbers: PX623032–4, PX623044–5, PV933159–61, PX623178–82, PX623200–1, and PX663655–6 (BioProject PRJNA1288426).

Gastropod Surveillance

In an additional effort to investigate the presence of *A. cantonensis* in intermediate hosts, 17 free-ranging slugs of 2 invasive species (15 *Ambigolimax valentianus* and 2 *Deroceras reticulatum*) were opportunistically collected from the wallaby enclosure and adjacent areas at the San Diego Zoo. The slugs were euthanized following a two-step method described previously using 5% and 70% ethanol, sequentially (*4*). Using tissues of slugs was determined to be exempt from Institutional Animal Care and Use Committee (IACUC) review via a Determination of Need form through the San Diego Zoo Wildlife Alliance's IACUC committee as the Public Health Service Policy on Humane Care and Use of Laboratory Animals and United States Department of Agriculture Animal Welfare Act do not cover the use of invertebrates.

While histopathology identified nematode larvae encysted in various tissues of a single *Ambigolimax valentianus*, the identity of these nematodes could not be confirmed despite various attempts of molecular characterization via conventional PCR with multiple primer sets and nanopore sequencing (data not shown). Therefore, the intermediate host(s) of *A. cantonensis* in San Diego still remains unknown. Reports of gastropod intermediate hosts in the continental United States are from Florida (*5–7*), Louisiana (*8*), and Georgia (*9*), and include multiple invasive species (*Alcadia striata*, *Bradybaena similaris*, *Cipangopaludina* spp., *Lissachatina fulica*, *Paropeas achatinaceum*, *Pomacea* spp., and *Zachrysia provisoria*) as well as native species (*Succinea floridana*, *Ventridens demissus*, and *Zonitoides arboreus*).

References

1. Apichat V, Narongrit S, Jittranuch T, Anucha W, Wilaiwan P, Chamaiporn F, et al. Phylogeny of *Angiostrongylus cantonensis* in Thailand based on cytochrome c oxidase subunit I gene sequence. Southeast Asian J Trop Med Public Health. 2016;47:377–86. PubMed
2. Rodpai R, Intapan PM, Thanchomnang T, Sanpool O, Sadaow L, Laymanivong S, et al. *Angiostrongylus cantonensis* and *A. malaysiensis* broadly overlap in Thailand, Lao PDR, Cambodia and Myanmar: a molecular survey of larvae in land snails. PLoS One. 2016;11:e0161128. PubMed https://doi.org/10.1371/journal.pone.0161128
3. Qvarnstrom Y, Sullivan JJ, Bishop HS, Hollingsworth R, da Silva AJ. PCR-based detection of *Angiostrongylus cantonensis* in tissue and mucus secretions from molluscan hosts. Appl Environ Microbiol. 2007;73:1415–9. PubMed https://doi.org/10.1128/AEM.01968-06
4. Gilbertson CR, Wyatt JD. Evaluation of euthanasia techniques for an invertebrate species, land snails (*Succinea putris*). J Am Assoc Lab Anim Sci. 2016;55:577–81. PubMed
5. Iwanowicz DD, Sanders LR, Schill WB, Xayavong MV, da Silva AJ, Qvarnstrom Y, et al. Spread of the rat lungworm (*Angiostrongylus cantonensis*) in giant African land snails (*Lissachatina fulica*) in Florida, USA. J Wildl Dis. 2015;51:749–53. PubMed https://doi.org/10.7589/2014-06-160
6. Stockdale Walden HD, Slapcinsky JD, Roff S, Mendieta Calle J, Diaz Goodwin Z, Stern J, et al. Geographic distribution of *Angiostrongylus cantonensis* in wild rats (*Rattus rattus*) and terrestrial snails in Florida, USA. PLoS One. 2017;12:e0177910. PubMed https://doi.org/10.1371/journal.pone.0177910
7. Stockdale-Walden HD, Slapcinsky J, Qvarnstrom Y, McIntosh A, Bishop HS, Rosseland B. *Angiostrongylus cantonensis* in introduced gastropods in southern Florida. J Parasitol. 2015;101:156–9. PubMed https://doi.org/10.1645/14-553.1
8. Teem JL, Qvarnstrom Y, Bishop HS, da Silva AJ, Carter J, White-McLean J, et al. The occurrence of the rat lungworm, *Angiostrongylus cantonensis*, in nonindigenous snails in the Gulf of Mexico region of the United States. Hawaii J Med Public Health. 2013;72(Suppl 2):11–4. PubMed
9. Achatz TJ, Chun CH, Young MA, Page J, Rowe M, Cooper C, et al. Detection of rat lungworms in invasive mollusks, Georgia, USA, 2024. Emerg Infect Dis. 2025;31:1852–4. PubMed https://doi.org/10.3201/eid3109.250133

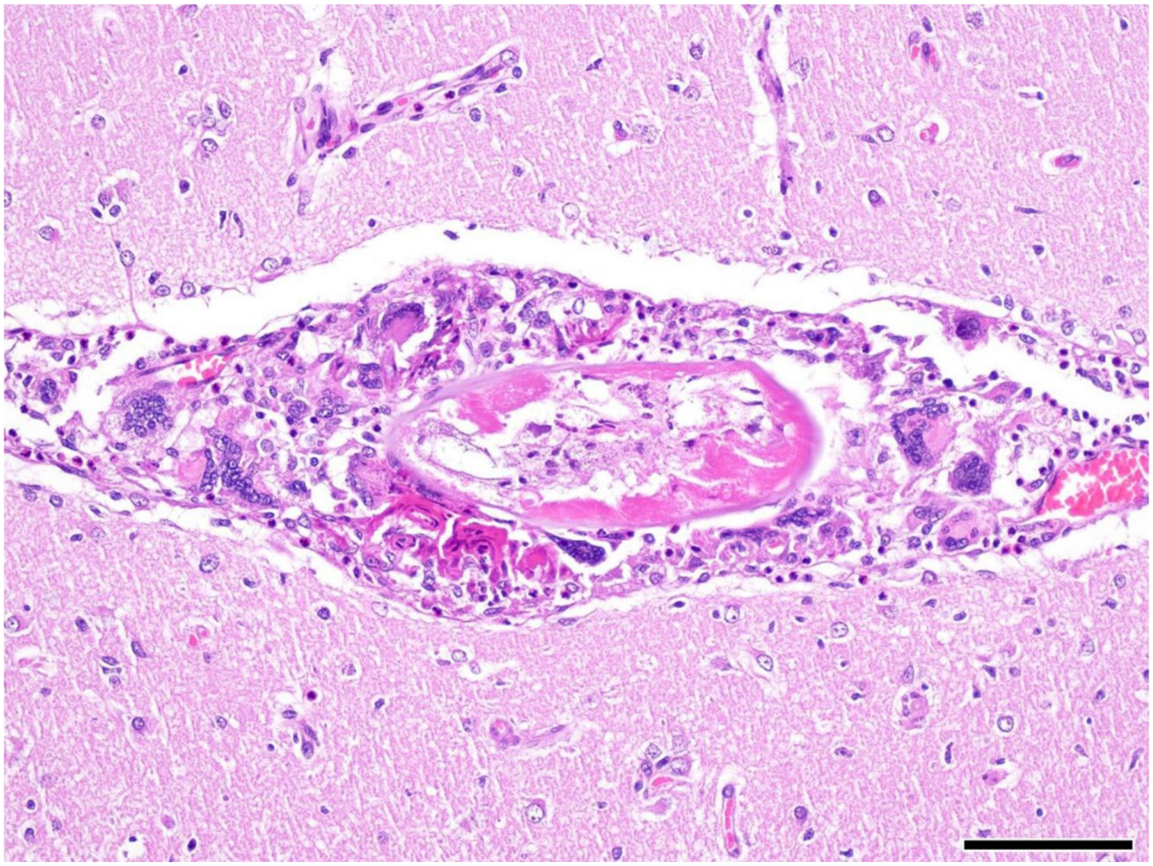

Appendix Figure. Brain, Virginia opossum (*Didelphis virginiana*). The leptomeninges is expanded by an eosinophilic and granulomatous infiltrate centered on a partially degenerate nematode with a smooth cuticle and coelomyarian-polymyarian musculature. Hematoxylin and eosin. Bar = 100 µm.
